# Pre-Target Interception Defines Carbapenem Failure in Carbapenem-Resistant Enterobacterales: A Mechanistic Framework for Spatiotemporal Drug Reprogramming

**DOI:** 10.3390/pharmaceutics18060717

**Published:** 2026-06-10

**Authors:** Eman Marzouk, Ayman Elbehiry

**Affiliations:** Department of Public Health, College of Applied Medical Sciences, Qassim University, P.O. Box 6666, Buraydah 51452, Saudi Arabia; e.marzouk@qu.edu.sa

**Keywords:** carbapenem-resistant enterobacterales (CRE), nanomedicine, nanoparticle drug delivery, Pre-Target Interception Model (PTIM), antibiotic resistance, drug distribution, biofilm, β-lactamases

## Abstract

Carbapenem-resistant Enterobacterales (CRE) are a major therapeutic challenge because of limited treatment options and high mortality. Despite advances in resistance-targeted therapies and pharmacokinetic (PK) optimization, treatment failure remains common. This review examines how resistance mechanisms and antibiotic exposure at the infection site jointly influence therapeutic outcomes in CRE infections. A mechanistic synthesis of evidence on carbapenem PKs, bacterial resistance, and nanoparticle (NP)-based delivery systems was performed. Based on this analysis, we propose the Pre-Target Interception Model (PTIM), which describes treatment failure as the progressive loss of active antibiotic before reaching penicillin-binding proteins. Unlike conventional approaches that focus primarily on resistance determinants or drug delivery platforms, PTIM emphasizes the factors that limit effective antibiotic exposure within infected tissues. Within this framework, nanocarrier systems are assessed according to their ability to protect antibiotics, enhance tissue penetration, and improve retention under conditions of enzymatic degradation, membrane restriction, efflux activity, and biofilm-associated diffusion barriers. However, clinical translation remains limited by manufacturing challenges, variability in NP performance, and the lack of validation in CRE-specific settings. Future progress will require quantitative measurement of antibiotic exposure at infection sites, standardized evaluation of nanocarrier performance, and validation in clinically relevant models. PTIM provides a framework for the rational development of nanomedicines designed to improve antibiotic delivery in CRE infections.

## 1. Introduction

Carbapenem-resistant Enterobacterales (CRE) are among the most serious antimicrobial resistance threats worldwide [1,2]. Enterobacterales comprise a large order of Gram-negative bacteria that includes clinically important pathogens such as *Klebsiella pneumoniae*, *Escherichia coli*, and *Enterobacter cloacae* complex. These organisms cause bloodstream, respiratory, urinary tract, and intra-abdominal infections, particularly in hospitalized and critically ill patients [3]. The global spread of CRE has been driven by horizontal transfer of mobile genetic elements carrying carbapenem resistance determinants [3]. CRE infections are associated with prolonged hospitalization, increased healthcare costs, and limited treatment options [4]. Carbapenem resistance is also linked to significantly higher mortality than that observed in infections caused by carbapenem-susceptible Enterobacterales [5,6]. Because of their clinical impact, carbapenem-resistant Gram-negative pathogens have been classified as critical-priority organisms by the World Health Organization [7].

Carbapenems remain essential for the treatment of severe infections caused by multidrug-resistant Gram-negative bacteria [8]. Their activity depends on penetration through outer membrane porins and binding to penicillin-binding proteins (PBPs), leading to inhibition of peptidoglycan synthesis and disruption of cell wall integrity [8]. However, the effectiveness of these agents has been progressively reduced by the emergence of multiple resistance mechanisms [3,8]. Carbapenemases, including KPC, NDM, VIM, IMP, and OXA-48-like enzymes, hydrolyze carbapenems before target interaction and represent the dominant resistance mechanism in many regions [3,8]. Additional mechanisms, including porin loss, reduced membrane permeability, efflux pump overexpression, and coexisting β-lactamases, further decrease intracellular antibiotic concentrations [8,9].

These mechanisms rarely act in isolation. Antibiotics encounter multiple barriers before reaching their targets [10]. They must cross host tissues, infection microenvironments, extracellular matrices, bacterial envelopes, and enzymatic defense systems [10,11]. Biofilms further restrict antibiotic activity by limiting penetration and creating spatially heterogeneous environments. Cells within biofilms adopt diverse metabolic states, including slow-growing and persistent phenotypes with reduced antimicrobial susceptibility [12]. Biofilm-associated tolerance therefore results from structural, physiological, and genetic factors rather than impaired diffusion alone [12]. These observations indicate that antimicrobial efficacy depends not only on resistance determinants but also on the processes that govern drug delivery and target accessibility [10,13].

The relationship between antimicrobial exposure and bacterial response is described by pharmacokinetic and pharmacodynamic (PK/PD) principles [14]. Antibacterial activity depends on drug exposure relative to pathogen susceptibility rather than administered dose alone [14,15]. For β-lactams, efficacy is closely linked to maintaining drug concentrations above the minimum inhibitory concentration (MIC) for sufficient durations [14]. Failure to achieve PK/PD targets is associated with reduced bacterial eradication and poorer clinical outcomes [15]. Consequently, PK/PD target attainment has become a cornerstone of antimicrobial dose optimization [14,15].

Achieving optimal exposure is particularly challenging in critically ill patients. Severe infection alters antibiotic distribution and elimination [16]. Increased capillary permeability, fluid resuscitation, hypoalbuminemia, organ dysfunction, extracorporeal support, and augmented renal clearance can substantially affect antimicrobial PKs [16,17]. As a result, patients receiving identical doses often achieve markedly different drug exposures [16].

Studies have repeatedly shown inadequate PK/PD target attainment with standard dosing regimens in critically ill populations [17]. These findings support the need for individualized therapeutic strategies. Dosing requirements may also vary according to the site of infection, because antibiotic penetration differs among bloodstream, pulmonary, urinary tract, and intra-abdominal compartments. Thus, route of administration and dosing strategy influence the likelihood of achieving effective drug exposure at the site of infection [16,17].

Despite advances in antimicrobial pharmacology, important limitations remain in the interpretation of treatment failure. Resistance-centered frameworks explain how bacteria reduce susceptibility, whereas PK/PD models describe exposure–response relationships and increasingly incorporate tissue exposure [14,15]. However, neither framework explicitly integrates the sequence of biological barriers that determine whether active antibiotic molecules ultimately reach their targets. Between administration and target engagement, antibiotics may be lost through altered tissue distribution, infection-site heterogeneity, biofilm-associated protection, permeability restrictions, enzymatic degradation, and active efflux [10,12,13]. These processes can reduce effective antibiotic exposure at the site of action despite adequate systemic concentrations and apparent in vitro susceptibility. Hence, treatment failure may result from cumulative pre-target losses occurring across host, microenvironmental, and bacterial compartments [10,13].

Recent advances have expanded treatment options for CRE. New β-lactam/β-lactamase inhibitor combinations and cefiderocol have improved outcomes against selected CRE populations [3,18,19]. Nevertheless, resistance to these agents continues to emerge, and therapeutic success remains influenced by both pathogen- and host-related factors [3]. Current management of CRE may also involve aminoglycosides, polymyxins, tigecycline, and combination regimens depending on pathogen susceptibility and infection site [3,19]. Nanocarrier-based delivery has also been investigated with multiple antimicrobial classes, although carbapenems remain the focus of this review because of their central role in CRE treatment and resistance. These findings suggest that effective therapy requires preservation of active antibiotic throughout the pathway leading to target engagement [10,11,13].

Nanomedicine offers a potential strategy to address these challenges. Nanoparticle (NP)-based delivery systems can protect antibiotics from premature degradation, improve localization within infected tissues, and provide controlled drug release [11,13]. Engineered nanocarriers can also respond to local microenvironmental conditions and overcome barriers that restrict antibiotic availability [11].

This narrative review was developed through searches of PubMed, Scopus, and Web of Science for literature published between January 2000 and March 2026. Search terms included combinations of “carbapenem-resistant Enterobacterales”, “CRE”, “carbapenem resistance”, “carbapenemases”, “carbapenem pharmacokinetics”, “biofilms”, “antimicrobial tolerance”, “nanocarriers”, “nanoparticles”, “liposomes”, “solid lipid nanoparticles”, “polymeric nanoparticles”, “nanomedicine”, and “drug delivery”. Studies related to CRE pathogenesis, carbapenem activity, resistance mechanisms, infection-site determinants of antibiotic exposure, and nanocarrier-based antimicrobial delivery were considered. Priority was given to peer-reviewed studies providing clinical, translational, mechanistic, or nanomedicine-related evidence relevant to CRE. Publications not directly related to CRE, carbapenem therapy, or antimicrobial nanomedicine were excluded. The selected literature was synthesized according to the major biological and pharmacological factors influencing carbapenem activity, including enzymatic degradation, permeability restriction, efflux activity, biofilm-associated barriers, and infection-site determinants of antibiotic exposure and therapeutic outcome.

**Figure 1:**
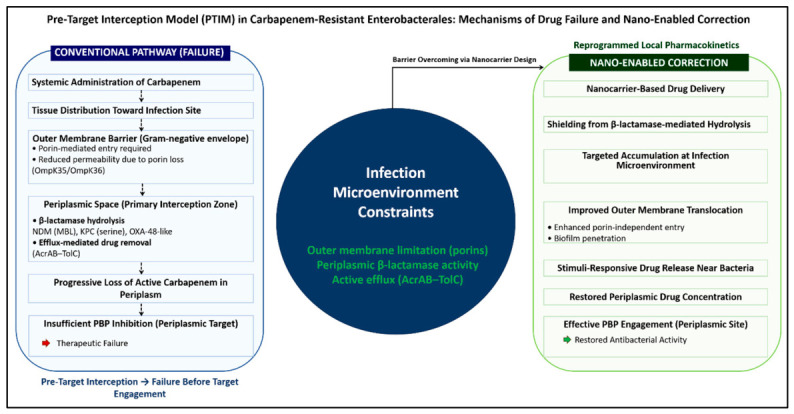
PTIM in CRE. Schematic representation of carbapenem failure in CRE and its correction through nanocarrier-based delivery. In the conventional pathway, restricted porin-mediated entry limits drug influx, while periplasmic β-lactamases hydrolyze active molecules and efflux systems remove residual drug. These processes reduce periplasmic concentration below the threshold required for penicillin-binding protein engagement, resulting in therapeutic failure. The infection microenvironment reinforces these barriers through permeability limitations, enzymatic degradation, biofilm-associated protection, and active efflux. In the nano-enabled pathway, nanocarriers protect the drug during transit, enhance localization at the infection site, and improve interaction with the bacterial envelope. Localized and stimuli-responsive release increases periplasmic drug concentration, enabling effective target engagement and restoration of antibacterial activity. The PTIM framework defines failure as pre-target loss and identifies restoration of local drug availability as the determinant of efficacy.

To integrate these concepts, this review introduces the Pre-Target Interception Model (PTIM). PTIM proposes that therapeutic success depends on preserving active antibiotic throughout the pathway leading to target engagement. Instead of focusing solely on resistance mechanisms or exposure metrics, PTIM examines how enzymatic degradation, permeability restrictions, efflux activity, biofilm-associated barriers, and infection-site heterogeneity collectively reduce effective antibiotic availability before target interaction [8,10,12]. Figure 1 illustrates this framework and highlights how cumulative pre-target losses contribute to carbapenem failure, whereas nanocarrier-based delivery may help preserve effective antibiotic exposure. This review examines the biological basis of pre-target interception in CRE, evaluates nanocarrier-based strategies designed to overcome these barriers, and discusses the opportunities and challenges associated with clinical translation.

## 2. Resistance Architecture in Enterobacterales: Spatial Control of Drug Fate

Carbapenem resistance in Enterobacterales is governed by a spatially structured system that determines antibiotic fate within the bacterial envelope. This system regulates entry, transformation, and persistence among defined compartments, establishing whether carbapenems reach PBPs at effective levels [20]. Evidence supports a layered organization in which enzymatic and non-enzymatic processes converge to limit intracellular availability [21].

### 2.1. Enzymatic Hydrolysis in the Periplasmic Space

Carbapenemases hydrolyze the β-lactam ring and eliminate activity prior to target binding. Major groups include class A serine enzymes (KPC), class B metallo-β-lactamases (NDM, VIM, IMP), and class D oxacillinases (OXA-48-like), which are widely distributed in CRE [22,23].

Resistance architecture varies among Enterobacterales species. *Klebsiella pneumoniae* producing *Klebsiella pneumoniae* carbapenemase (KPC) commonly combines carbapenemase production with porin alterations, whereas *Escherichia coli* producing New Delhi metallo-β-lactamase (NDM) frequently relies on plasmid-mediated dissemination of carbapenem resistance determinants. In the *Enterobacter cloacae* complex, carbapenem resistance may involve interactions among permeability defects, AmpC overexpression, and acquired carbapenemases. These differences indicate that the relative contribution of individual resistance mechanisms is species-dependent and may influence therapeutic response [23,24]. These enzymes localize within the periplasm or at the outer membrane interface, positioning catalytic activity directly along the influx pathway and enabling immediate degradation following membrane translocation [22,25].

Metallo-β-lactamases exhibit broad substrate profiles and resistance to current inhibitors. Certain variants associate with the outer membrane or are released in vesicles, extending hydrolytic activity beyond the producing cell and reducing surrounding drug availability [22,23]. Hydrolysis acts in coordination with permeability limitation and efflux, forming an early interception step that reduces active drug before accumulation [24].

### 2.2. Restricted Entry Through Outer Membrane Remodeling

The outer membrane regulates antibiotic influx into Gram-negative bacteria. Carbapenems access the periplasm through porin channels [26]. Alterations in porin expression or structure, particularly OmpK35 and OmpK36 in *K. pneumoniae*, reduce permeability and limit translocation. These changes result from gene disruption, structural mutation, or transcriptional regulation and decrease intracellular accumulation [24,27].

Membrane remodeling also involves changes in lipopolysaccharide composition and lipid organization, which alter membrane properties and further restrict diffusion [26]. Reduced influx becomes significant when combined with hydrolysis or efflux, positioning permeability as a modulatory component within the resistance system [27].

### 2.3. Active Efflux as a Clearance Mechanism

Efflux systems reduce intracellular antibiotic levels through active transport. In Enterobacterales, resistance-nodulation-division transporters such as AcrAB–TolC span the envelope and mediate direct extrusion into the external environment. Mutations in regulatory elements, including acrR, increase pump expression and enhance export capacity [28].

Efflux limits exposure duration at the site of action. For β-lactams, activity depends on sustained presence, and rapid removal reduces interaction with PBPs [3]. This mechanism operates with permeability reduction and enzymatic degradation, forming a coordinated system that restricts entry and accelerates clearance [28]. Among CRE isolates, carbapenemase production remains the dominant resistance determinant, whereas permeability defects and efflux systems generally act as complementary mechanisms that further reduce intracellular drug exposure and enhance resistance levels [3,24].

### 2.4. Biofilm-Induced Diffusion Barriers

Biofilm formation contributes to persistence and reduced treatment response. Clinical data show frequent coexistence of biofilm formation and carbapenemase production in *Klebsiella pneumoniae* isolates [29]. The extracellular matrix, composed of polysaccharides, proteins, and extracellular DNA, restricts diffusion and delays antibiotic penetration. Interactions with matrix components reduce the free fraction of drug and lower effective concentrations before reaching bacterial cells [30].

Biofilms also generate gradients in nutrients and oxygen, producing heterogeneous populations. Cells in deeper layers exhibit reduced metabolic activity, which decreases susceptibility to β-lactams that require active cell wall synthesis [30]. This barrier extends resistance beyond individual cells by maintaining protected subpopulations under limited exposure [29].

### 2.5. Coordinated Interception Network

Carbapenem resistance emerges from the combined effect of mechanisms that regulate antibiotic flux across compartments. Carbapenemase production, permeability reduction, and efflux frequently coexist in CRE. Their interaction creates a continuous interception pathway in which antibiotics are excluded, degraded, or removed prior to target interaction. Synergy between reduced entry and enzymatic hydrolysis establishes the dominant resistance phenotype, with efflux further decreasing availability [21,31].

Experimental and clinical observations reveal that resistance is often greatest when carbapenemase production occurs together with permeability defects or enhanced efflux activity. This cooperative behavior supports the PTIM concept that therapeutic failure results from cumulative pre-target losses rather than the action of a single resistance mechanism alone [21,24,31].

These processes operate across space and time, requiring successful membrane passage, persistence within the periplasm, and sufficient duration for target interaction. Disruption at any stage reduces efficacy. Within the PTIM framework, this system represents pre-target control of drug availability. Failure reflects inability to achieve effective local concentration at PBPs despite systemic exposure [21]. Table 1 summarizes the spatial localization of key resistance components in CRE and their specific impact on carbapenem fate before target engagement.

## 3. Mechanistic Basis of Therapeutic Failure: The PTIM Framework

### 3.1. Defining the Carbapenem Journey

Carbapenems exert bactericidal activity by reaching PBPs and inhibiting cell wall synthesis [32,33]. Their efficacy depends on maintaining adequate exposure over time and achieving sufficient drug concentrations at the site of action [32,34]. In CRE infections, therapeutic outcome is influenced not only by systemic exposure but also by events occurring during drug transit through tissues, infection microenvironments, and bacterial compartments [35]. Therefore, antibacterial activity depends on the balance between drug delivery and cumulative loss before target engagement. The PTIM framework examines how these sequential loss processes influence the amount of active drug that ultimately reaches PBPs.

### 3.2. Early Loss of Active Drug Through Hydrolysis

Carbapenemases hydrolyze the β-lactam ring and eliminate antibacterial activity before target binding [34,36]. Within the PTIM framework, enzymatic degradation represents a pre-target loss process because active drug molecules may be inactivated before interacting with PBPs. The periplasmic localization of many carbapenemases places hydrolysis directly along the influx pathway, creating competition between target access and drug destruction [37]. As a result, therapeutic success depends not only on the amount of drug delivered to the infection site but also on the fraction that remains active after transit through the periplasmic compartment. KPC, NDM, and OXA-type carbapenemases expand substrate coverage and substantially reduce the probability of successful target engagement [34,36,37].

### 3.3. Limited Intracellular Exposure

Reduced permeability and active efflux influence the same pharmacological endpoint: intracellular drug exposure. Carbapenems depend on porin-mediated entry, and structural alterations in outer membrane channels reduce antibiotic influx [31,35,38]. Efflux systems further decrease intracellular concentrations by actively exporting antibiotics after entry [35,39]. Although these mechanisms are often considered separately, their combined effect is a reduction in both the amount and duration of active drug available for target interaction. Within PTIM, restricted influx and accelerated efflux therefore function as complementary determinants of pre-target drug loss that can amplify the impact of enzymatic degradation and further limit effective target exposure [38,39].

### 3.4. Misalignment with Infection Microenvironments

Effective therapy requires alignment between drug distribution and bacterial localization. In CRE infections, heterogeneous infection microenvironments can disrupt this relationship [35,40]. Biofilms create structured environments in which extracellular matrix components and restricted diffusion reduce antibiotic penetration into deeper bacterial populations [33,41]. Subsequently, bacteria occupying different regions within the same infection may experience markedly different drug exposures despite identical systemic dosing. This spatial mismatch between drug distribution and bacterial localization creates areas of suboptimal exposure and represents an additional source of pre-target drug loss within the PTIM framework [42,43].

### 3.5. PTIM as a CRE-Specific Model

Carbapenem resistance emerges through the combined action of enzymatic degradation, restricted influx, active efflux, and infection-site barriers that collectively reduce active drug availability before target engagement [35,38,39]. Experimental and clinical observations indicate that resistance is often greatest when carbapenemase production occurs together with permeability defects or enhanced efflux activity, supporting the concept that therapeutic failure results from cumulative pre-target losses rather than the action of a single mechanism alone [21,24,31].

**Figure 2:**
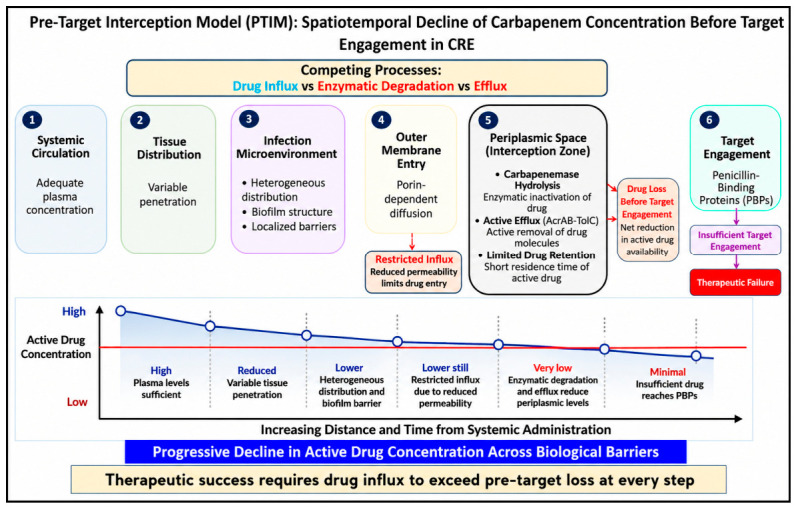
Spatiotemporal decline of carbapenem concentration in CRE. Following administration, drug levels decrease within tissue, infection microenvironments, and bacterial compartments due to restricted entry, periplasmic enzymatic degradation, and active efflux. These processes reduce exposure below thresholds required for PBP engagement, resulting in insufficient target inhibition and therapeutic failure.

PTIM proposes that therapeutic failure in CRE can be viewed through the lens of progressive pre-target drug loss. Rather than replacing established molecular resistance mechanisms, the model integrates these processes within a spatiotemporal framework centered on drug delivery to the site of action. Therapeutic success therefore requires that local drug influx exceeds cumulative loss from degradation, restricted permeability, efflux activity, and diffusion barriers before target interaction can occur [14,15,16]. Figure 2 illustrates the PTIM framework by linking sequential reductions in carbapenem concentration to restricted entry, enzymatic degradation, efflux activity, and infection-site barriers that ultimately reduce target engagement in CRE.

PTIM is presented as a conceptual framework derived from established PK, microbiological, and resistance evidence. Direct quantitative validation of cumulative pre-target carbapenem loss across biological compartments remains limited. Future studies combining spatial imaging, PK modeling, and compartment-specific drug measurements will be required to determine the extent to which PTIM predicts therapeutic outcomes in CRE infections.

## 4. Nanocarrier Platforms for CRE Therapy

The treatment of CRE remains challenging because resistance mechanisms frequently coexist with biofilm formation, reduced membrane permeability, efflux pump activity, and enzymatic antibiotic degradation. These factors limit antibiotic penetration and compromise therapeutic efficacy despite the availability of newer antimicrobial agents. Nanocarrier-based delivery systems have therefore emerged as promising strategies to improve antimicrobial PKs, enhance bacterial targeting, facilitate biofilm penetration, and increase local drug concentrations at sites of infection [10,11,13]. The major nanocarrier platforms currently investigated for CRE therapy, together with their advantages, limitations, and representative applications, are summarized in Table 2.

### 4.1. Liposomes

Liposomes are phospholipid vesicles capable of encapsulating both hydrophilic and hydrophobic antimicrobial agents. Their biocompatibility, ability to protect antibiotics from premature degradation, and capacity for controlled drug release have made them one of the most extensively investigated antimicrobial nanocarriers [44,45]. Liposomal encapsulation can prolong circulation time, improve biodistribution, and enhance antibiotic accumulation at sites of infection while facilitating interaction with bacterial membranes and biofilms [46].

Despite these advantages, liposomes may exhibit limited storage stability, drug leakage during prolonged storage, and relatively complex manufacturing requirements [47]. Nevertheless, their favorable safety profile and extensive clinical experience continue to support their development for antimicrobial delivery.

### 4.2. Polymeric NPs

Polymeric NPs are among the most versatile antimicrobial delivery systems because their physicochemical characteristics, release kinetics, and surface properties can be precisely engineered. Biodegradable polymers such as chitosan, poly(lactic-co-glycolic acid) (PLGA), and polycaprolactone (PCL) have demonstrated considerable potential for antibiotic delivery [48,49].

These systems improve antibiotic stability, prolong antimicrobial exposure, and facilitate sustained drug release. In addition, polymeric NPs can enhance penetration through biofilm matrices and increase intracellular drug accumulation, thereby addressing several mechanisms associated with phenotypic tolerance [50].

Abdelkader et al. [51] demonstrated that meropenem-loaded chitosan NPs exhibited enhanced antibacterial activity against multidrug-resistant *Klebsiella pneumoniae* and significantly increased bacterial clearance and survival in a septic animal model compared with free meropenem. Similarly, Shaaban et al. [52] reported improved antibacterial activity of imipenem-loaded polymeric NPs against resistant Gram-negative pathogens.

More recently, Ebrahimi et al. [53] developed imipenem-loaded PCL NPs against carbapenem-resistant *K. pneumoniae*. The formulation significantly enhanced antibacterial activity, inhibited biofilm formation, and reduced expression of *bla_OXA-48_*, *bla_NDM_*, and *bla_IMP_* resistance genes. These findings suggest that polymeric NPs may simultaneously improve antibiotic delivery and interfere with resistance-associated bacterial responses.

The primary limitations of polymeric NPs include formulation complexity, scale-up challenges, and manufacturing variability [54,55]. Nonetheless, they currently represent one of the most promising nanocarrier platforms for CRE-directed therapy.

### 4.3. Solid Lipid NPs

Solid lipid NPs (SLNs) combine the advantages of lipid-based systems with improved physical stability and controlled-release characteristics. Their solid lipid matrix protects encapsulated compounds from degradation and supports prolonged antimicrobial exposure [56].

Nazari and Hosseini [57] demonstrated that gentamicin-loaded SLNs significantly enhanced antibacterial activity against multidrug-resistant *Acinetobacter baumannii*. The formulation reduced minimum inhibitory concentrations, improved biofilm inhibition, and suppressed expression of the biofilm-associated bap gene. Similarly, Nemattalab et al. [58] reported enhanced antibacterial and antibiofilm activity of cinnamon oil-loaded SLNs against multidrug-resistant *Escherichia coli*.

These studies illustrate that SLNs can improve antimicrobial delivery while simultaneously targeting biofilm-associated virulence mechanisms. However, drug-loading limitations and the relative scarcity of CRE-specific investigations remain important barriers to broader application.

### 4.4. Metallic NPs

Metallic NPs differ from conventional nanocarriers because they possess intrinsic antimicrobial activity. Silver NPs (AgNPs) are the most extensively studied and exert antibacterial effects through membrane disruption, oxidative stress generation, protein dysfunction, and structural damage to bacterial cells [59,60].

Gondal et al. [61] reported synergistic antibacterial activity between silver NPs and meropenem against carbapenem-resistant Enterobacterales, suggesting that NP-antibiotic combinations may partially restore susceptibility to existing antimicrobial agents. Similarly, Wozeak et al. [62] demonstrated that biogenic AgNPs inhibited growth and biofilm formation in carbapenem-resistant *Klebsiella pneumoniae* through disruption of membrane integrity and biofilm architecture.

Although metallic NPs exhibit potent antimicrobial activity, concerns regarding cytotoxicity, biodistribution, long-term accumulation, and environmental impact remain major obstacles to clinical translation [63,64].

### 4.5. Hybrid Nanocarrier Systems

Hybrid nanocarriers integrate complementary characteristics from multiple nanoplatforms to achieve enhanced therapeutic performance. By combining lipid and polymer components, these systems can simultaneously optimize drug loading, stability, release kinetics, bacterial targeting, and biofilm penetration [65,66].

Baek et al. [65] developed lipid-polymer hybrid NPs that demonstrated sustained antibiotic release, strong biofilm binding, and significant reductions in biofilm viability at concentrations substantially lower than those required for free antibiotics. Additional evidence has been provided by nanoemulsion-based systems co-delivering meropenem and bioactive compounds, which enhanced antibacterial activity against carbapenem-resistant *Klebsiella pneumoniae* [67].

The principal advantage of hybrid systems lies in their ability to address multiple resistance-associated barriers simultaneously. However, formulation complexity, reproducibility concerns, and regulatory challenges remain important obstacles to clinical translation [55,68].

### 4.6. Comparative Perspective and Relevance to PTIM

The major strengths and limitations of currently available nanocarrier platforms are summarized in Table 2. Liposomes primarily improve membrane interaction and drug protection, polymeric NPs offer tunable release and surface engineering, SLNs emphasize formulation stability and prolonged exposure, metallic NPs contribute intrinsic antimicrobial activity, whereas hybrid systems combine multiple delivery functions within a single platform.

**Figure 3:**
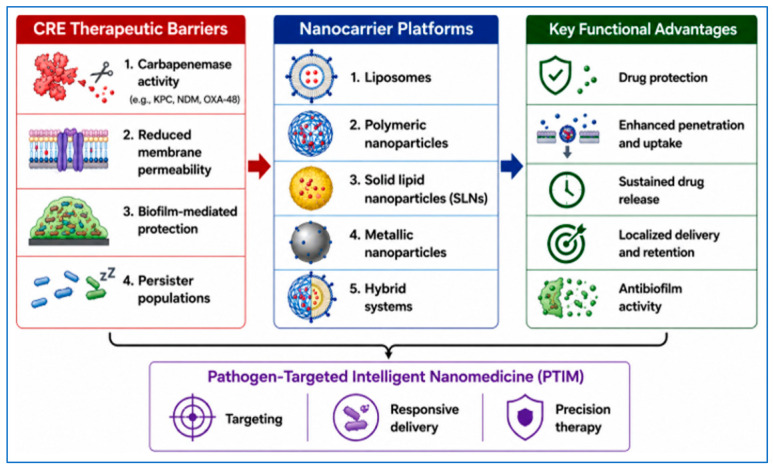
Conceptual framework illustrating how nanocarrier platforms address key therapeutic barriers in CRE infections and support the transition toward PTIM.

Significantly, these nanocarriers address several biological barriers that contribute to therapeutic failure in CRE infections, including impaired antibiotic penetration, biofilm-associated tolerance, and reduced local drug exposure [12]. As shown in Figure 3, nanocarrier systems can strengthen antibiotic stability, facilitate penetration through biofilm matrices, enhance intracellular delivery, and increase antimicrobial concentrations at sites of infection [69,70].

However, conventional nanocarriers primarily function as delivery platforms and do not fully address the biological complexity of CRE infections. Biofilm-associated heterogeneity, metabolic adaptation, dynamic infection microenvironments, and resistance-mediated drug inactivation continue to compromise treatment outcomes [71]. Thus, increasing attention has shifted toward multifunctional nanomedicines capable of combining targeted recognition, infection-responsive activation, antibiofilm activity, and precision antimicrobial delivery [72].

These developments provide the conceptual foundation for PTIM, which extends beyond conventional nanocarriers by integrating adaptive targeting and microenvironment-responsive therapeutic functions [73,74]. Despite these advances, most nanocarrier systems remain passive delivery vehicles and lack the adaptive recognition and responsiveness required to address the dynamic nature of CRE infections. The following section discusses PTIM strategies and their potential role in overcoming the complex biological barriers that characterize CRE infections.

**Table 2:** Key characteristics of nanocarrier platforms investigated for antimicrobial delivery against CRE.

Platform	Major Advantages	Major Limitations	Carbapenem Compatibility	Representative Encapsulation Efficiency (%)	Refs.
Liposomes	Accommodate hydrophilic and hydrophobic agents; biocompatible; facilitate membrane interaction and biofilm penetration	Drug leakage; limited long-term stability	Meropenem and combination formulations can be efficiently incorporated	94% (meropenem-loaded liposomes); 86% (AgNP-incorporated meropenem liposomes)	[75]
Polymeric NPs	Tunable release; surface functionalization; protection of labile drugs	Manufacturing complexity; scale-up challenges	Successfully loaded with meropenem and imipenem	76.3% (meropenem–chitosan NPs); 84.5% (imipenem–PCL NPs); 88.3% (meropenem–chitosan/TPP NPs)	[51,53,76]
Metallic NPs	Intrinsic antimicrobial activity; antibiofilm effects	Potential toxicity and tissue accumulation	Frequently combined with carbapenems to enhance antibacterial activity	72% (imipenem-loaded AuNPs); 74% (meropenem-loaded AuNPs)	[61,62,77]
SLNs/NLCs	Sustained release; enhanced stability; protection of labile compounds	Limited loading capacity; lipid polymorphism	Suitable for carbapenem encapsulation and controlled release	85.7% (meropenem-loaded NLCs); 89.9% (meropenem-loaded SLNs)	[78,79]
Hybrid Systems	Combine targeting, controlled release, and barrier penetration functions	Formulation complexity; regulatory challenges	Compatible with combination antimicrobial delivery	Formulation-dependent; insufficient comparative data available	[65,67]

Reported encapsulation efficiencies varied according to carrier composition and formulation strategy, ranging from 72% for carbapenem-loaded gold NPs to 94% for meropenem-loaded liposomes. Polymeric NPs achieved encapsulation efficiencies of 76.3–88.3%, whereas lipid-based systems, including SLNs and NLCs, reached 85.7–89.9%, indicating that both polymeric and lipid-based platforms can effectively encapsulate carbapenems [51,53,75,76,77,79]. Platform selection is also influenced by infection site, required tissue penetration, and intended route of administration, factors that remain insufficiently evaluated in CRE-specific studies.

Among currently investigated platforms, polymeric NPs and lipid-based systems (SLNs/NLCs) have shown the most consistent performance for carbapenem delivery because they combine high encapsulation efficiency with controlled release and protection of labile antibiotics. However, available evidence remains insufficient to support a single optimal platform across all infection types and clinical settings.

## 5. PTIM-Guided Nanocarrier Functions in CRE Therapy

Nanocarriers influence antibiotic performance by modifying events that occur before drug-target interaction. Rather than directly reversing resistance, these systems improve drug preservation, distribution, release, and access to bacterial populations. Their role is to increase the probability that active antibiotic molecules reach bacterial targets despite enzymatic degradation, permeability barriers, efflux activity, and biofilm-associated diffusion limitations [80].

### 5.1. Drug Protection and Controlled Exposure

Premature antibiotic degradation reduces the fraction of active drug available at the infection site. Encapsulation protects antibiotics during transit and limits exposure to conditions that promote drug loss before bacterial contact [81]. Liposomal and polymeric systems improve stability by shielding antibiotics during distribution [44,47]. Polymeric NPs carrying carbapenems have demonstrated enhanced antibacterial activity against resistant Gram-negative pathogens, partly through preservation of drug integrity during delivery [48,53].

Current evidence suggests that polymeric NPs and lipid-based systems achieve high carbapenem encapsulation efficiencies, typically ranging from 76% to 90%, whereas metallic NP formulations generally show lower loading efficiencies but may offer additional antimicrobial activity [53,77,79].

Release kinetics further influence local antibiotic availability. Stimuli-responsive carriers release their cargo in response to infection-associated signals, including pH changes and enzymatic activity, whereas sustained-release systems prolong local exposure and reduce rapid concentration decline [10,11,13]. However, protection during transit does not eliminate resistance mechanisms. Following release, carbapenems remain susceptible to degradation by carbapenemases located within bacterial compartments [82,83].

### 5.2. Localization and Barrier Navigation

Effective therapy requires antibiotic accumulation at sites where bacterial populations reside. Nanocarriers improve retention within infected tissues and increase local drug concentrations relative to free antibiotics [50,69]. For example, gentamicin-loaded solid lipid NPs demonstrated enhanced antibiofilm and antibacterial activity against multidrug-resistant *Acinetobacter baumannii*, while carbapenem-loaded polymeric NPs improved antibacterial activity against resistant Gram-negative pathogens by increasing local drug delivery [53,57]. Their interactions with extracellular structures and bacterial surfaces support distribution within heterogeneous infection environments that are often poorly accessible to conventional therapy [60,84].

At the bacterial interface, NP properties such as size, surface charge, and functionalization influence membrane interactions and may facilitate access across permeability barriers [85]. These effects can improve antibiotic delivery to Gram-negative bacteria and partially reduce limitations imposed by restricted membrane permeability [41,72,86]. Nevertheless, membrane interactions are not fully selective and may also affect host cells, creating safety considerations that require careful optimization [87].

### 5.3. Biofilm Penetration and Intracellular Availability

Biofilms represent a major obstacle in CRE infections because dense extracellular matrices restrict antibiotic diffusion and create physiologically diverse bacterial populations [71,88]. Nanocarriers can improve transport through these structures and increase antibiotic delivery to embedded cells [59,89].

Enhanced antibiofilm activity has been reported for polymeric, lipid-based, and multifunctional systems against resistant pathogens, including CRE [53,90,91]. Representative examples include meropenem-loaded chitosan NPs and lipid-based formulations, which showed increased activity against resistant Gram-negative bacteria and enhanced penetration into biofilm-associated populations [53,91].

Nanocarriers may also increase intracellular antibiotic exposure by delivering concentrations that temporarily exceed efflux capacity [92]. However, active efflux systems remain functional and continue to export antibiotics after delivery [70]. As a result, intracellular accumulation can be transient unless sustained exposure is maintained.

Experimental studies suggest that nanocarrier-mediated delivery can transiently increase intracellular antibiotic concentrations and improve antibacterial activity despite the presence of active efflux systems. However, these effects generally reflect increased local drug exposure rather than direct inhibition of efflux pumps. Thus, sustained therapeutic benefit depends on maintaining intracellular concentrations above export-mediated losses over time [70,92].

### 5.4. Multifunctional Nanocarriers and PTIM Relevance

Recent nanocarrier designs combine multiple delivery functions within a single platform, including drug protection, targeted localization, membrane interaction, biofilm penetration, and controlled release [41]. Hybrid systems integrate complementary mechanisms to improve delivery across multiple biological barriers simultaneously [56,64,93]. Surface functionalization can further enhance antibacterial and antibiofilm activity against CRE pathogens [94]. Functionalization with targeting ligands can promote interaction with bacterial surfaces, biofilm components, or infected tissues, thereby improving local drug accumulation. However, targeting efficiency remains variable because NP distribution is influenced by protein adsorption, immune recognition, and clearance by phagocytic cells.

Despite these advances, increasing formulation complexity introduces challenges related to reproducibility, stability, manufacturing, and regulatory evaluation [95]. Accordingly, greater functional sophistication does not necessarily translate into improved clinical performance.

Within the PTIM framework, nanocarriers are best viewed as systems that modify pre-target events rather than directly neutralize resistance mechanisms. Their principal contribution lies in reducing cumulative drug loss before target engagement. As summarized in Table 3, nanocarrier systems can improve drug preservation, localization, penetration, and retention, thereby increasing the likelihood that effective antibiotic concentrations reach bacterial targets despite persistent resistance determinants.

## 6. Design Constraints in CRE-Targeted Nanomedicine

The therapeutic performance of nanocarriers is determined by a series of competing design requirements. Properties that improve bacterial targeting, retention, and delivery may simultaneously increase toxicity, instability, immune recognition, or manufacturing complexity. These limitations arise from fundamental NP characteristics, including size, surface charge, composition, and structural organization, all of which influence biological behavior and therapeutic performance [63,101,102,103]. Thus, optimization requires balancing multiple parameters rather than maximizing a single property [104].

### 6.1. Biological Design Trade-Offs

Successful delivery depends on efficient interaction with bacterial surfaces while maintaining acceptable host safety. Surface properties that promote interaction with the lipopolysaccharide-rich outer membrane of Gram-negative bacteria may also increase interactions with mammalian cells [105,106]. Experimental studies have revealed that highly reactive NPs can induce oxidative stress, membrane damage, and inflammatory responses, particularly when particle size is reduced or surface reactivity is increased [107,108,109]. Effective design therefore requires sufficient bacterial interaction without exceeding toxicity thresholds [63,106].

A second challenge involves balancing stability and drug release. Nanocarriers must remain sufficiently stable during circulation to preserve drug integrity, yet release their cargo efficiently after reaching the infection site [110,111]. Stimuli-responsive systems attempt to address this challenge by linking release to environmental signals such as pH changes or enzymatic activity [112,113]. Nonetheless, infection sites often exhibit considerable biological variability, which may result in incomplete, delayed, or premature drug release [114]. Hence, stability and responsiveness frequently act in opposing directions, limiting uniform performance across diverse infection environments [115,116].

Targeting introduces an additional trade-off. Surface modification can improve localization and increase interaction with bacterial or host structures [10,73]. At the same time, altered surface characteristics may promote recognition by the mononuclear phagocyte system, leading to accelerated clearance from circulation [117,118]. Strategies designed to reduce immune recognition often rely on surface shielding, which prolongs circulation but may reduce targeting efficiency by altering NP interactions within biological environments [119,120]. Hence, prolonged circulation and precise localization are often difficult to achieve simultaneously [74,121].

### 6.2. Formulation and Translation Trade-Offs

Drug loading capacity strongly influences therapeutic efficacy but also affects NP stability. Increasing payload can enhance local antibiotic concentrations; however, excessive loading may destabilize carrier structure, alter release behavior, and promote premature leakage [122,123,124]. Conversely, insufficient loading may fail to achieve effective concentrations at the infection site. Formulation design therefore requires balancing payload capacity with structural integrity and controlled release characteristics [11,122].

System complexity presents a further challenge. Multifunctional nanocarriers often combine targeting ligands, responsive release mechanisms, and barrier-navigation capabilities within a single platform [54,55]. Although these features may improve biological performance, they also increase formulation sensitivity, batch variability, manufacturing demands, and regulatory complexity [102,125]. Simpler systems generally offer greater reproducibility and scalability, whereas highly engineered formulations may provide broader functionality but face greater obstacles during clinical development [126,127].

Clinical translation illustrates the impact of these trade-offs. Many antimicrobial nanoformulations show promising in vitro activity but do not progress beyond preclinical development because of stability limitations, manufacturing challenges, limited scalability, insufficient in vivo efficacy, or regulatory barriers [55,126]. Similar challenges have slowed the clinical adoption of multifunctional nanocarrier systems despite encouraging preclinical results [55,127]. These observations highlight the gap between formulation optimization and successful clinical translation.

**Figure 4:**
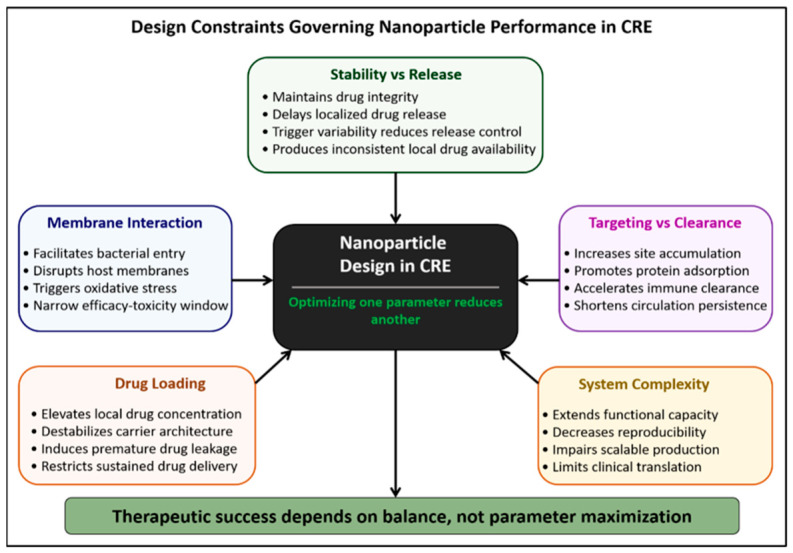
Competing design constraints governing NP performance in CRE. NP performance is defined by interdependent properties among biological interfaces. Membrane interaction enhances bacterial entry but increases host toxicity. Stability preserves drug integrity but delays release. Surface functionalization improves localization while promoting immune clearance. Increased payload raises concentration but destabilizes structure and accelerates leakage. System complexity expands functionality but reduces reproducibility and scalability. These competing effects prevent simultaneous optimization and require balanced design.

The principal challenge in CRE-targeted nanomedicine is therefore not the optimization of a single characteristic, but the integration of multiple competing requirements within a clinically practical system. Table 4 summarizes the major design constraints influencing NP performance, while Figure 4 illustrates the interconnected trade-offs that govern delivery efficiency, safety, stability, and translational feasibility.

## 7. Translational Barriers in CRE Nanotherapy

The clinical translation of nanocarrier-based therapies remains challenging despite substantial advances in formulation design. In CRE infections, therapeutic success depends not only on drug delivery but also on achieving sufficient antibiotic concentrations at bacterial targets after sequential losses caused by permeability barriers, enzymatic degradation, efflux activity, and biofilm-associated diffusion limitations [39]. However, current development frameworks primarily rely on systemic PK measurements that do not adequately reflect antibiotic availability at sites of infection [16,17].

A major limitation is the lack of site-specific PK data. Following administration, nanocarriers undergo continuous transformation through interactions with plasma proteins, immune components, and biological matrices, generating heterogeneous distribution patterns that cannot be predicted from plasma concentrations alone [128,129]. This challenge becomes more pronounced in biofilm-associated infections, where restricted diffusion and matrix binding reduce the freely available fraction of antibiotic and create concentration gradients across the biofilm structure [46,130]. Therefore, improved circulation time or tissue accumulation does not necessarily indicate restoration of effective target exposure because antibacterial activity depends on local drug concentrations at the site of action rather than systemic levels [131]. Delivery improvement therefore remains uncoupled from therapeutic outcome.

Manufacturing represents a second barrier to translation. Nanocarrier performance is highly dependent on formulation parameters, and small variations in particle size, surface composition, or drug loading may alter biological behavior and therapeutic activity [54,132]. These variations can lead to batch-to-batch inconsistency and reduced reproducibility during scale-up [68,133]. Maintaining consistent nanoscale architecture across production cycles remains difficult, particularly when moving from laboratory preparation to industrial manufacturing [134,135,136,137].

Experience from the broader nanomedicine field further illustrates the difficulty of clinical translation. Numerous multifunctional NP systems have demonstrated promising preclinical activity but have not progressed to routine clinical use because increasing formulation complexity often compromises reproducibility, scalability, and regulatory evaluation [126,127]. In contrast, clinically successful nanomedicines are generally based on simpler and more reproducible platforms, emphasizing the importance of balancing functionality with translational feasibility.

Regulatory assessment presents an additional challenge. Nanocarriers exhibit dynamic physicochemical properties and context-dependent biological behavior that are not fully captured by evaluation frameworks originally developed for conventional pharmaceuticals [55,126]. Comprehensive characterization of particle structure, drug release behavior, biodistribution, and long-term safety is therefore required before clinical implementation.

Safety considerations remain incompletely understood. Nanocarriers often accumulate in organs involved in clearance, including the liver and spleen, where biological responses depend on particle composition, surface properties, and duration of exposure [74,117]. Potential risks include immune activation, tissue retention, and off-target effects, highlighting the need for extended safety evaluation.

Clinical evidence specific to CRE infections remains limited. Most studies have been conducted in vitro or in animal models, while controlled clinical investigations involving CRE pathogens are scarce [10,35]. Human PK data, long-term safety assessments, and evidence demonstrating improved clinical outcomes remain insufficient. Economic factors also influence translation, as complex synthesis procedures, specialized materials, and advanced characterization requirements increase development costs and may limit accessibility in regions with a high CRE burden [54,55].

Several approaches may help address these barriers. Standardized characterization protocols, harmonized reporting of particle properties and release behavior, and improved manufacturing controls may enhance reproducibility across studies [55,126]. Advances in imaging, biodistribution analysis, and site-specific PK assessment may also improve evaluation of local drug exposure within infected tissues. These developments could support more reliable translation of nanocarrier systems into clinical CRE applications.

Overall, these challenges highlight a central gap between experimental success and clinical implementation. Nanocarriers can improve drug distribution and modify pre-target events, but their ability to restore effective antibiotic exposure at sites of infection remains difficult to verify under clinical conditions [15]. Progress toward clinical application will require direct measurement of local drug exposure, reproducible large-scale manufacturing, regulatory frameworks adapted to nanomedicine, and well-designed clinical studies focused on CRE infections [16,126]. Until these requirements are addressed, nanotherapy for CRE should be considered a promising but still emerging therapeutic strategy.

## 8. Future Directions and Perspective

### 8.1. Future Research Priorities

The next stage of nanomedicine development for CRE infections should move beyond improving antibiotic delivery and focus on demonstrating measurable therapeutic benefit at sites of infection. Although numerous nanocarrier platforms have shown promising preclinical activity, clinical translation remains limited because local drug exposure, instead of systemic concentration, ultimately determines antibacterial efficacy [15,16,17]. Future studies should therefore prioritize methods capable of directly quantifying antibiotic concentrations within infected tissues, biofilms, and bacterial microenvironments.

Advances in responsive nanocarriers may provide new opportunities to improve treatment precision. Systems that respond to infection-associated signals, including enzymatic activity, pH variation, or inflammatory mediators, have the potential to synchronize drug release with local disease conditions and reduce unnecessary drug exposure in noninfected tissues [83,138]. However, the performance of these systems must be validated under clinically relevant conditions that reflect the biological complexity of CRE infections.

Improved characterization of drug distribution within biofilms and structured tissues is another important priority. Biofilm-associated concentration gradients remain poorly defined despite their central role in treatment failure [12]. Integrating advanced imaging technologies, spatial PK analyses, and infection-site monitoring may improve understanding of how antibiotics and nanocarriers behave within heterogeneous infection environments.

Future delivery systems should also address the combined influence of permeability barriers, enzymatic degradation, efflux activity, and biofilm-associated diffusion limitations. Rather than targeting individual resistance mechanisms in isolation, emerging approaches are likely to benefit from multifunctional designs that integrate protection, localization, controlled release, and sustained exposure within a single platform [10,41,94].

Equally important is the generation of clinically relevant evidence. Standardized experimental models, reproducible manufacturing strategies, and well-designed clinical studies are required to establish the therapeutic value of PTIM-guided nanomedicine [55,126]. Comparative studies against current standards of care, together with long-term safety and PK evaluations, will be essential for determining whether improved delivery translates into meaningful clinical benefit [126,127].

Ultimately, the future of CRE nanotherapy lies in the transition from empirical drug delivery toward precision antimicrobial intervention. Within the PTIM framework, this goal requires the integration of spatial PKs, infection-responsive delivery, and quantitative assessment of local drug exposure [16,17,116]. Achieving these objectives may enable the development of nanomedicine platforms capable of delivering effective antibiotic concentrations precisely where bacterial survival and resistance emerge [10].

### 8.2. Strengths and Limitations of PTIM

The PTIM offers several conceptual strengths for understanding therapeutic failure in CRE infections. First, it integrates bacterial resistance mechanisms with drug distribution processes, providing a unified framework that links antibiotic exposure to target engagement [15,16]. Second, PTIM introduces a spatial perspective by emphasizing that antibacterial efficacy depends on drug availability at the site of action rather than systemic concentrations alone [16,17]. Third, the framework is inherently delivery-oriented and therefore provides a useful basis for evaluating nanocarrier strategies designed to reduce cumulative drug loss before target interaction [10].

Despite these advantages, several limitations should be recognized. PTIM remains a conceptual framework and has not yet been experimentally validated across diverse CRE infection models. Furthermore, the relative contribution of individual loss processes may vary according to pathogen characteristics, infection site, host factors, and antibiotic class [39]. Direct assessment of PTIM predictions also requires site-specific PK measurements, which remain technically challenging in many infection settings [16,17]. Accordingly, PTIM should be viewed as a hypothesis-generating framework that can guide future experimental and translational research rather than as a validated predictor of clinical outcome. Further studies integrating local drug exposure measurements with microbiological and clinical endpoints will be required to determine its utility in antimicrobial development [35,126].

## 9. Conclusions

Carbapenem-resistant Enterobacterales (CRE) remain a major therapeutic challenge because multiple resistance mechanisms reduce active antibiotic concentrations before effective target engagement occurs. This review highlights how the Pre-Target Interception Model (PTIM) provides a complementary framework for understanding treatment failure by emphasizing drug availability at the site of action as a critical determinant of therapeutic success. Within this framework, enzymatic degradation, permeability restriction, efflux activity, and biofilm-associated diffusion collectively reduce the fraction of active antibiotic reaching penicillin-binding proteins. Nanocarrier systems offer several opportunities to modify antibiotic delivery through drug protection, localized accumulation, controlled release, enhanced biofilm penetration, and prolonged exposure within infected tissues. However, these systems do not eliminate intrinsic resistance mechanisms and continue to face biological, engineering, and translational constraints. Polymeric and lipid-based systems demonstrate efficient carbapenem delivery, but no universally optimal platform has been identified. Major barriers to clinical translation include limited site-specific PK data, manufacturing reproducibility, regulatory evaluation, long-term safety assessment, and the scarcity of CRE-focused clinical studies. Future progress will depend on integrating advanced nanocarrier design with quantitative assessment of local drug exposure, standardized evaluation strategies, and rigorous validation in clinically relevant CRE models. Overall, PTIM provides a mechanistic framework for guiding the development of precision antimicrobial delivery systems. Future success in CRE nanomedicine will depend not only on improving antibiotic delivery but also on demonstrating sustained therapeutic concentrations at the site of action under clinically relevant conditions.

## Figures and Tables

**Table 1:** Resistance components, spatial localization, and impact on carbapenem fate in CRE.

Resistance Component	Primary Spatial Location	Mechanistic Action	Impact on Carbapenem Fate	Refs.
Carbapenemases (KPC, NDM, OXA-48-like)	Periplasm and outer membrane interface	β-lactam ring hydrolysis	Loss of activity before PBP interaction	[22,23,25]
Porin alterations (OmpK35, OmpK36)	Outer membrane	Loss or modification of diffusion channels	Reduced entry and limited accumulation	[24,26,27]
Membrane remodeling	Outer membrane	Lipid and structural changes	Decreased permeability and diffusion	[26]
Efflux pumps (AcrAB–TolC)	Envelope-spanning system	Active export of antibiotics	Reduced exposure duration at target site	[3]
Efflux regulation (e.g., acrR)	Cytoplasmic regulatory level	Increased pump expression	Enhanced drug removal	[28]
Biofilm matrix	Extracellular environment	Diffusion restriction and binding	Delayed penetration and reduced free drug	[30]
Biofilm metabolic states	Biofilm interior	Reduced growth activity	Lower susceptibility to β-lactams	[30]
Integrated resistance architecture	Multiple compartments	Combined action of all mechanisms	Failure to reach effective concentration at PBPs	[21]

**Table 3:** Nanocarrier functions mapped to pre-target barriers in CRE.

Nanocarrier Function	Targeted Pre-Target Barrier	Mechanistic Action	Effect on Carbapenem Fate	Refs.
Drug protection during systemic transit	Early enzymatic degradation before bacterial contact	Encapsulation isolates antibiotic from β-lactamases	Preserves active fraction before reaching infection site	[44,47,48,49,81]
Tissue-level accumulation at infection sites	Spatial mismatch between distribution and bacterial localization	Retention in inflamed environments increases local concentration	Elevates drug density beyond diffusion limits	[50,60,69,84]
Environment-triggered release near bacteria	Premature dispersion before bacterial contact	Stimuli-responsive release under infection conditions	Increases availability proximal to bacterial cells	[10,11,13,96]
Membrane interaction and biofilm penetration	Restricted entry and diffusion limitation	Membrane disruption and matrix penetration	Improves access to periplasm and embedded populations	[39,59,71,88,89,97]
Prolongation of local exposure	Rapid clearance and insufficient exposure duration	Controlled release and enhanced retention	Maintains concentrations above required thresholds	[17,45,96,98,99,100]

**Table 4:** Design constraints governing NP performance in CRE.

Design Constraint	Functional Role	Limiting Mechanism	Translational Consequence	Refs.
Membrane interaction	Enables bacterial envelope interaction	Non-selective interaction damages host membranes and induces oxidative stress	Limits concurrent optimization of penetration and safety	[63,106,108]
Stability versus release	Preserves drug during circulation	Excess stability delays release; trigger variability disrupts timing	Prevents alignment between persistence and effective exposure	[110,115,116]
Targeting versus clearance	Enhances localization	Protein adsorption increases immune recognition and clearance	Limits sustained accumulation at infection sites	[74,118,121]
Drug loading versus structure	Increases local concentration	High payload destabilizes structure and promotes leakage	Prevents stable delivery under high drug demand	[11,122,123]
System complexity versus scalability	Integrates multiple functions	Formulation sensitivity and variability reduce reproducibility	Limits large-scale production and clinical consistency	[55,126,127]

## Data Availability

No new data were created or analyzed in this study.

## References

[1] Chen H.-Y., Jean S.-S., Lee Y.-L., Lu M.-C., Ko W.-C., Liu P.-Y., Hsueh P.-R. (2021). Carbapenem-resistant Enterobacterales in long-term care facilities: A global and narrative review. Front. Cell. Infect. Microbiol..

[2] Caliskan-Aydogan O., Alocilja E.C. (2023). A review of carbapenem resistance in Enterobacterales and its detection techniques. Microorganisms.

[3] Tompkins K., Van Duin D. (2021). Treatment for carbapenem-resistant Enterobacterales infections: Recent advances and future directions. Eur. J. Clin. Microbiol. Infect. Dis..

[4] Hassoun-Kheir N., Hussien K., Karram M., Saffuri M., Badaan S., Peleg S., Aboelhega W., Warman S., Alon T., Pollak D. (2023). Clinical significance and burden of carbapenem-resistant Enterobacterales (CRE) colonization acquisition in hospitalized patients. Antimicrob. Resist. Infect. Control.

[5] Martin A., Fahrbach K., Zhao Q., Lodise T. (2018). Association between carbapenem resistance and mortality among adult, hospitalized patients with serious infections due to Enterobacteriaceae: Results of a systematic literature review and meta-analysis. Open Forum Infect. Dis..

[6] Kohler P.P., Volling C., Green K., Uleryk E.M., Shah P.S., McGeer A. (2017). Carbapenem resistance, initial antibiotic therapy, and mortality in *Klebsiella pneumoniae* bacteremia: A systematic review and meta-analysis. Infect. Control Hosp. Epidemiol..

[7] World Health Organization (WHO) (2024). WHO Bacterial Priority Pathogens List, 2024: Bacterial Pathogens of Public Health Importance, to Guide Research, Development and Strategies to Prevent and Control Antimicrobial Resistance.

[8] Papp-Wallace K.M., Endimiani A., Taracila M.A., Bonomo R.A. (2011). Carbapenems: Past, present, and future. Antimicrob. Agents Chemother..

[9] Kim S., Kim S.R., Xuan X., Park Y., Roh S.J., Kim S. (2024). Prevalence of Carbapenem-Resistant Enterobacterales and Their Diverse Resistance Mechanisms. Biomed. Sci. Lett..

[10] Zou W., McAdorey A., Yan H., Chen W. (2023). Nanomedicine to overcome antimicrobial resistance: Challenges and prospects. Nanomedicine.

[11] Torchilin V.P. (2014). Multifunctional, stimuli-sensitive nanoparticulate systems for drug delivery. Nat. Rev. Drug Discov..

[12] Hall C.W., Mah T.-F. (2017). Molecular mechanisms of biofilm-based antibiotic resistance and tolerance in pathogenic bacteria. FEMS Microbiol. Rev..

[13] Gao W., Chen Y., Zhang Y., Zhang Q., Zhang L. (2018). Nanoparticle-based local antimicrobial drug delivery. Adv. Drug Deliv. Rev..

[14] Craig W.A. (1998). Pharmacokinetic/pharmacodynamic parameters: Rationale for antibacterial dosing of mice and men. Clin. Infect. Dis..

[15] Drusano G.L. (2004). Antimicrobial pharmacodynamics: Critical interactions of’bug and drug’. Nat. Rev. Microbiol..

[16] Roberts J.A., Lipman J. (2009). Pharmacokinetic issues for antibiotics in the critically ill patient. Crit. Care Med..

[17] Abdul-Aziz M.H., Alffenaar J.-W.C., Bassetti M., Bracht H., Dimopoulos G., Marriott D., Neely M.N., Paiva J.-A., Pea F., Sjovall F. (2020). Antimicrobial therapeutic drug monitoring in critically ill adult patients: A Position Paper# MH Abdul-Aziz et al. Intensive Care Med..

[18] Sader H.S., Mendes R.E., Duncan L., Kimbrough J.H., Carvalhaes C.G., Castanheira M. (2023). Ceftazidime-avibactam, meropenem-vaborbactam, and imipenem-relebactam activities against multidrug-resistant Enterobacterales from United States Medical Centers (2018–2022). Diagn. Microbiol. Infect. Dis..

[19] Bonnin R.A., Bernabeu S., Emeraud C., Naas T., Girlich D., Jousset A.B., Dortet L. (2023). In vitro activity of imipenem-relebactam, meropenem-vaborbactam, ceftazidime-avibactam and comparators on carbapenem-resistant non-carbapenemase-producing Enterobacterales. Antibiotics.

[20] Reygaert W.C. (2018). An overview of the antimicrobial resistance mechanisms of bacteria. AIMS Microbiol..

[21] Miftode I.-L., Radu V.D., Jigoranu R.-A., Leca D.-A., Prepeliuc C.S., Pasare M.A., Miftode R.-S., Grigoriu M.G., Parângă T.G., Miftode E.G. (2026). Novel Insights into Carbapenem Resistance: Mechanisms, Diagnostics, and Future Directions. Antibiotics.

[22] Alvisi G., Curtoni A., Fonnesu R., Piazza A., Signoretto C., Piccinini G., Sassera D., Gaibani P. (2025). Epidemiology and genetic traits of carbapenemase-producing enterobacterales: A global threat to human health. Antibiotics.

[23] Hansen G.T. (2021). Continuous evolution: Perspective on the epidemiology of carbapenemase resistance among Enterobacterales and other Gram-negative bacteria. Infect. Dis. Ther..

[24] Baroud Á., Dandache I., Araj G., Wakim R., Kanj S., Kanafani Z., Khairallah M., Sabra A., Shehab M., Dbaibo G. (2013). Underlying mechanisms of carbapenem resistance in extended-spectrum β-lactamase-producing *Klebsiella pneumoniae* and *Escherichia coli* isolates at a tertiary care centre in Lebanon: Role of OXA-48 and NDM-1 carbapenemases. Int. J. Antimicrob. Agents.

[25] Aurilio C., Sansone P., Barbarisi M., Pota V., Giaccari L.G., Coppolino F., Barbarisi A., Passavanti M.B., Pace M.C. (2022). Mechanisms of action of carbapenem resistance. Antibiotics.

[26] Zdarska V., Arcari G., Kolar M., Mlynarcik P. (2026). Antibiotic Resistance in *Klebsiella pneumoniae* and Related Enterobacterales: Molecular Mechanisms, Mobile Elements, and Therapeutic Challenges. Antibiotics.

[27] Alizadeh N., Ahangarzadeh Rezaee M., Samadi Kafil H., Hasani A., Soroush Barhaghi M.H., Milani M., Yeganeh Sefidan F., Memar M.Y., Lalehzadeh A., Ghotaslou R. (2020). Evaluation of resistance mechanisms in carbapenem-resistant Enterobacteriaceae. Infect. Drug Resist..

[28] Tristancho-Baró A., López-Calleja A.I., Milagro A., Ariza M., Viñeta V., Fortuño B., López C., Latorre-Millán M., Clusa L., Badenas-Alzugaray D. (2025). Mechanisms of cefiderocol resistance in carbapenemase-producing enterobacterales: Insights from comparative genomics. Antibiotics.

[29] El Naggar N.M., Shawky R.M., Serry F.M., Emara M. (2024). Investigating the relationship between carbapenemase production and biofilm formation in *Klebsiella pneumoniae* clinical isolates. BMC Res. Notes.

[30] Belay W.Y., Getachew M., Tegegne B.A., Teffera Z.H., Dagne A., Zeleke T.K., Abebe R.B., Gedif A.A., Fenta A., Yirdaw G. (2024). Mechanism of antibacterial resistance, strategies and next-generation antimicrobials to contain antimicrobial resistance: A review. Front. Pharmacol..

[31] Başaran S.N., Öksüz L. (2025). Newly developed antibiotics against multidrug-resistant and carbapenem-resistant Gram-negative bacteria: Action and resistance mechanisms. Arch. Microbiol..

[32] Kato T., Yagi Y., Maruyama T., Hamada Y. (2025). Pharmacokinetics/Pharmacodynamics-Based Repositioning of Cefmetazole and Flomoxef in Extended-Spectrum β-Lactamase-Producing Enterobacterales Treatment: An Injectable Carbapenem-Sparing and Outpatient Strategy. Antibiotics.

[33] Uddin T.M., Chakraborty A.J., Khusro A., Zidan B.R.M., Mitra S., Emran T.B., Dhama K., Ripon M.K.H., Gajdács M., Sahibzada M.U.K. (2021). Antibiotic resistance in microbes: History, mechanisms, therapeutic strategies and future prospects. J. Infect. Public Health.

[34] Huang Z., Bian X., Li Y., Hu J., Guo B., Yang X., Jin Y., Zheng S., Wang X., Gao C. (2024). In vitro pharmacokinetics/pharmacodynamics of FL058 (a novel beta-lactamase inhibitor) combined with meropenem against carbapenemase-producing Enterobacterales. Front. Pharmacol..

[35] Xu W., Shang L., Li X., Yu J., Shen J., Li Y., He Q., Chen L., Wang D., Wei Y. (2026). Current progress in antibacterial agents for carbapenem-resistant enterobacterales. Front. Pharmacol..

[36] Peaper D.R., Kulkarni M.V., Tichy A.N., Jarvis M., Murray T.S., Hodsdon M.E. (2013). Rapid detection of carbapenemase activity through monitoring ertapenem hydrolysis in Enterobacteriaceae with LC–MS/MS. Bioanalysis.

[37] Bush K., Bradford P.A. (2016). β-Lactams and β-lactamase inhibitors: An overview. Cold Spring Harb. Perspect. Med..

[38] van Loon K., Voor In ‘t holt A.F., Vos M.C. (2018). A systematic review and meta-analyses of the clinical epidemiology of carbapenem-resistant Enterobacteriaceae. Antimicrob. Agents Chemother..

[39] Li X.-Z., Plésiat P., Nikaido H. (2015). The challenge of efflux-mediated antibiotic resistance in Gram-negative bacteria. Clin. Microbiol. Rev..

[40] Mancuso G., De Gaetano S., Midiri A., Zummo S., Biondo C. (2023). The challenge of overcoming antibiotic resistance in carbapenem-resistant gram-negative bacteria:“Attack on Titan”. Microorganisms.

[41] Thakur A., Ganesan R., Dutta J.R. (2025). Nanomaterials Against Antimicrobial Resistance and Beyond: Toward Mitigating Bacterial Resuscitation. Total Environ. Microbiol..

[42] Xu J., Liu J., Zhao J., Tian T., Wang M., Yuan G., Peng Y., Zhang Y., Li Z., Kan B. (2024). Clonal and horizontal transmission of carbapenem-resistant Enterobacterales strains and genes via flies. Gut Pathog..

[43] Cartagena A.J., Taylor K.L., Lopez L.C., Su J., Smith J.T., Manson A.L., Chen J.D., Pierce V.M., Earl A.M., Bhattacharyya R.P. (2025). The carbapenem inoculum effect provides insights into the molecular mechanisms underlying carbapenem resistance in the Enterobacterales. mBio.

[44] Drulis-Kawa Z., Dorotkiewicz-Jach A. (2010). Liposomes as delivery systems for antibiotics. Int. J. Pharm..

[45] Allen T.M., Cullis P.R. (2013). Liposomal drug delivery systems: From concept to clinical applications. Adv. Drug Deliv. Rev..

[46] Ferraz M.P. (2024). Advanced nanotechnological approaches for biofilm prevention and control. Appl. Sci..

[47] Sercombe L., Veerati T., Moheimani F., Wu S.Y., Sood A.K., Hua S. (2015). Advances and challenges of liposome assisted drug delivery. Front. Pharmacol..

[48] Xiong M.-H., Bao Y., Yang X.-Z., Zhu Y.-H., Wang J. (2014). Delivery of antibiotics with polymeric particles. Adv. Drug Deliv. Rev..

[49] Kalhapure R.S., Suleman N., Mocktar C., Seedat N., Govender T. (2015). Nanoengineered drug delivery systems for enhancing antibiotic therapy. J. Pharm. Sci..

[50] Jiang L., Ding L., Liu G. (2023). Nanoparticle formulations for therapeutic delivery, pathogen imaging and theranostic applications in bacterial infections. Theranostics.

[51] Abdelkader A., El-Mokhtar M.A., Abdelkader O., Hamad M.A., Elsabahy M., El-Gazayerly O.N. (2017). Ultrahigh antibacterial efficacy of meropenem-loaded chitosan nanoparticles in a septic animal model. Carbohydr. Polym..

[52] Shaaban M.I., Shaker M.A., Mady F.M. (2017). Imipenem/cilastatin encapsulated polymeric nanoparticles for destroying carbapenem-resistant bacterial isolates. J. Nanobiotechnol..

[53] Ebrahimi F.A., Siasi E., Yazdian F., Ashrafi F. (2025). Nanotechnology Meets superbugs: Biocompatible polymeric nanoparticles combat MDR *Klebsiella pneumoniae* via gene suppression and biofilm Inhibition. Sci. Rep..

[54] Paliwal R., Babu R.J., Palakurthi S. (2014). Nanomedicine scale-up technologies: Feasibilities and challenges. AAPS PharmSciTech.

[55] Hua S., De Matos M.B., Metselaar J.M., Storm G. (2018). Current trends and challenges in the clinical translation of nanoparticulate nanomedicines: Pathways for translational development and commercialization. Front. Pharmacol..

[56] Mengesha Y. (2025). Nanomedicine approaches to enhance the effectiveness of meropenem: A strategy to tackle antimicrobial resistance. Discov. Nano.

[57] Nazari M., Hosseini S.M. (2025). Evaluation of antibacterial and antibiofilm efficacy of gentamicin-loaded solid lipid nanoparticles (GM-SLNs) against Acinetobacter baumannii infections. BMC Chem..

[58] Nemattalab M., Rohani M., Evazalipour M., Hesari Z. (2022). Formulation of Cinnamon (Cinnamomum verum) oil loaded solid lipid nanoparticles and evaluation of its antibacterial activity against Multi-drug Resistant *Escherichia coli*. BMC Complement. Med. Ther..

[59] Gupta A., Mumtaz S., Li C.-H., Hussain I., Rotello V.M. (2019). Combatting antibiotic-resistant bacteria using nanomaterials. Chem. Soc. Rev..

[60] Barani M., Zeeshan M., Kalantar-Neyestanaki D., Farooq M.A., Rahdar A., Jha N.K., Sargazi S., Gupta P.K., Thakur V.K. (2021). Nanomaterials in the management of gram-negative bacterial infections. Nanomaterials.

[61] Gondal A.J., Choudhry N., Bukhari H., Rizvi Z., Yasmin N. (2022). Characterization of genomic diversity among carbapenem-resistant *Escherichia coli* clinical isolates and antibacterial efficacy of silver nanoparticles from Pakistan. Microorganisms.

[62] Wozeak D.R., Pereira I.L., Cardoso T.L., Panagio L.A., Nakazato G., Acosta I.B., Varela A.S., Hartwig D.D. (2026). Biogenic Silver Nanoparticles: An Antibacterial and Antibiofilm Approach to Control Carbapenem-Resistant *Klebsiella pneumoniae*. Curr. Microbiol..

[63] Damasco J.A., Ravi S., Perez J.D., Hagaman D.E., Melancon M.P. (2020). Understanding nanoparticle toxicity to direct a safe-by-design approach in cancer nanomedicine. Nanomaterials.

[64] El-Khawaga A.M., Orlandini M., Raucci L., Elmaghraby K. (2025). Magnetic nanoparticles as a promising antimicrobial agent for combating multidrug resistant bacteria: A review. Discov. Appl. Sci..

[65] Baek J.-S., Tan C.H., Ng N.K.J., Yeo Y.P., Rice S.A., Loo S.C.J. (2018). A programmable lipid-polymer hybrid nanoparticle system for localized, sustained antibiotic delivery to Gram-positive and Gram-negative bacterial biofilms. Nanoscale Horiz..

[66] Wu S., Wei Y., Wang Y., Zhang Z., Liu D., Qin S., Shi J., Shen J. (2024). Liposomal antibiotic booster potentiates carbapenems for combating NDMs-producing *Escherichia coli*. Adv. Sci..

[67] Tang Y., Yang C., Liu C., Xu Y., Peng M., Chan E.W.-C., Chen S. (2024). Development of an effective meropenem/KPC-2 inhibitor combination to combat infections caused by carbapenem-resistant *Klebsiella pneumoniae*. Int. J. Antimicrob. Agents.

[68] Herdiana Y. (2025). Bridging the gap: The role of advanced formulation strategies in the clinical translation of nanoparticle-based drug delivery systems. Int. J. Nanomed..

[69] Hussain S., Joo J., Kang J., Kim B., Braun G.B., She Z.-G., Kim D., Mann A.P., Mölder T., Teesalu T. (2018). Antibiotic-loaded nanoparticles targeted to the site of infection enhance antibacterial efficacy. Nat. Biomed. Eng..

[70] Li M., Liu Y., Gong Y., Yan X., Wang L., Zheng W., Ai H., Zhao Y. (2023). Recent advances in nanoantibiotics against multidrug-resistant bacteria. Nanoscale Adv..

[71] Høiby N., Bjarnsholt T., Moser C., Bassi G., Coenye T., Donelli G., Hall-Stoodley L., Holá V., Imbert C., Kirketerp-Møller K. (2015). ESCMID guideline for the diagnosis and treatment of biofilm infections 2014. Clin. Microbiol. Infect..

[72] AlQurashi D.M., AlQurashi T.F., Alam R.I., Shaikh S., Tarkistani M.A.M. (2025). Advanced nanoparticles in combating antibiotic resistance: Current innovations and future directions. J. Nanotheranostics.

[73] Radovic-Moreno A.F. (2013). Bacteria-Targeting Nanoparticles for Managing Infections. Ph.D. Thesis.

[74] Blanco E., Shen H., Ferrari M. (2015). Principles of nanoparticle design for overcoming biological barriers to drug delivery. Nat. Biotechnol..

[75] Iqtedar M., Aslam M., Abdullah R., Kaleem A. (2025). Nanosomes carrying mycosynthesized silver nanoparticles as a drug delivery tool against MDR *Salmonella typhi* BTCB170. Eur. Chem. Biotechnol. J..

[76] Amini M.S., Baseri Salehi M., Bahador N. (2024). Evaluating the antibacterial effect of meropenem-loaded chitosan/sodium tripolyphosphate (TPP) nanoparticles on *Acinetobacter baumannii* isolated from hospitalized patients. BMC Infect. Dis..

[77] Shaker M.A., Shaaban M.I. (2017). Formulation of carbapenems loaded gold nanoparticles to combat multi-antibiotic bacterial resistance: In vitro antibacterial study. Int. J. Pharm..

[78] Wilson C.E. (2024). Development and Evaluation of Meropenem Encapsulated Nanostructured Lipid Carriers as an Antimicrobial Treatment of *Pseudomonas aeruginosa*. Master’s Thesis.

[79] Mhango E.K., Kalhapure R.S., Jadhav M., Sonawane S.J., Mocktar C., Vepuri S., Soliman M., Govender T. (2017). Preparation and optimization of meropenem-loaded solid lipid nanoparticles: In vitro evaluation and molecular modeling. AAPS PharmSciTech.

[80] Suswati E., Hibatulloh M.F., Putri D.E., Amini S.A. (2025). Comparative Effectiveness of Single, Dual, and Multi-Antibiotic Therapies in Managing Carbapenem-Resistant Enterobacterales: A Systematic Review and Meta-Analysis on Survival Rates. Trends Sci..

[81] Abdallah O.M., Shebl H.R., Abdelsalam E., Mehrez S.I. (2024). The impact and safety of encapsulated nanomaterials as a new alternative against carbapenem resistant bacteria. A systematic review. World J. Microbiol. Biotechnol..

[82] Jeon J.H., Lee J.H., Lee J.J., Park K.S., Karim A.M., Lee C.-R., Jeong B.C., Lee S.H. (2015). Structural basis for carbapenem-hydrolyzing mechanisms of carbapenemases conferring antibiotic resistance. Int. J. Mol. Sci..

[83] Lohans C.T., Freeman E.I., Groesen E.v., Tooke C.L., Hinchliffe P., Spencer J., Brem J., Schofield C.J. (2019). Mechanistic insights into β-lactamase-catalysed carbapenem degradation through product characterisation. Sci. Rep..

[84] Hayat S., Ashraf A., Siddique M.H., Aslam B., Shafaqat H., Javed S., Taj Z., Sarfraz M.H., Rafiq H., Muzammil S. (2025). Nanoparticle-mediated approaches to combat antibiotic resistance: A comprehensive review on current progress, mechanisms, and future perspectives. RSC Adv..

[85] Modi S.K., Gaur S., Sengupta M., Singh M.S. (2023). Mechanistic insights into nanoparticle surface-bacterial membrane interactions in overcoming antibiotic resistance. Front. Microbiol..

[86] Elbehiry A., Abalkhail A. (2025). Antimicrobial nanoparticles against superbugs: Mechanistic insights, biomedical applications, and translational frontiers. Pharmaceuticals.

[87] Canales C.S.C., Cazorla J.I.M., Cazorla R.M.M., Sábio R.M., Santos H.A., Pavan F.R. (2025). Combating Gram-negative infections: The role of antimicrobial peptides and nanotechnology in overcoming antibiotic resistance. Mater. Today Bio.

[88] Flemming H.-C., Wingender J., Szewzyk U., Steinberg P., Rice S.A., Kjelleberg S. (2016). Biofilms: An emergent form of bacterial life. Nat. Rev. Microbiol..

[89] Mohamed A.A., Saed S., El-Sayed S.R., Yassin M.T., Gad M., Tartour E., Fathey H.A., Taha A.S., Mohamed A.H., Al-Otibi F.O. (2024). A combined therapy of meropenem–ZnO nanoparticles efficiently eliminates carbapenem-resistant *Klebsiella pneumoniae* biofilms, with reduced nephrotoxicity (in vitro). Lett. Appl. Microbiol..

[90] Saffari Natanzi A., Poudineh M., Karimi E., Khaledi A., Haddad Kashani H. (2025). Innovative approaches to combat antibiotic resistance: Integrating CRISPR/Cas9 and nanoparticles against biofilm-driven infections. BMC Med..

[91] Elashkar E., Alfaraj R., El-Borady O.M., Amer M.M., Algammal A.M., El-Demerdash A.S. (2025). Novel silver nanoparticle-based biomaterials for combating *Klebsiella pneumoniae* biofilms. Front. Microbiol..

[92] Mouzakis A., Panagopoulos P., Papazoglou D., Petrakis V. (2025). A comprehensive review of nanoparticles in the fight against antimicrobial resistance. Pathogens.

[93] Elbehiry A., Alajaji A.I. (2026). Next-Generation Strategies for Controlling Foodborne Pathogens: Precision Antimicrobials, Biofilm Disruption, and Emerging Molecular Interventions. Foods.

[94] Pan J., Zhang J., Hu P., Yao Z., Zhang X., Zhou T., Shen M. (2025). Daidzein-Decorated Gold Nanoparticles as a Novel Antimicrobial Strategy Against Carbapenem-Resistant Enterobacteriaceae. Int. J. Nanomed..

[95] Angeles Flores G., Cusumano G., Venanzoni R., Angelini P. (2025). Advancements in antibacterial therapy: Feature papers. Microorganisms.

[96] Pelgrift R.Y., Friedman A.J. (2013). Nanotechnology as a therapeutic tool to combat microbial resistance. Adv. Drug Deliv. Rev..

[97] Huh A.J., Kwon Y.J. (2011). “Nanoantibiotics”: A new paradigm for treating infectious diseases using nanomaterials in the antibiotics resistant era. J. Control. Release.

[98] Roberts J.A., Abdul-Aziz M.H., Lipman J., Mouton J.W., Vinks A.A., Felton T.W., Hope W.W., Farkas A., Neely M.N., Schentag J.J. (2014). Individualised antibiotic dosing for patients who are critically ill: Challenges and potential solutions. Lancet Infect. Dis..

[99] Ventola C.L. (2015). The antibiotic resistance crisis: Part 1: Causes and threats. Pharm. Ther..

[100] Zhang L., Pornpattananangkul D., Hu C.-M., Huang C.-M. (2010). Development of nanoparticles for antimicrobial drug delivery. Curr. Med. Chem..

[101] Ly P.-D., Ly K.-N., Phan H.-L., Nguyen H.H., Duong V.-A., Nguyen H.V. (2024). Recent advances in surface decoration of nanoparticles in drug delivery. Front. Nanotechnol..

[102] El-Sayed Ellakwa D., Ellakwa T.E. (2025). Biocompatible nanoparticles in medicine: From design to clinical translation. Discov. Mater..

[103] Edo G.I., Ali A.B., Okolie M.C., Orogu J.O., Emumejaye K., Oghroro E.E.A., Jikah A.N., Yousif E., Igbuku U.A., Owheruo J.O. (2026). Toxicity of nanostructures in drug delivery applications. Pharm. Sci. Adv..

[104] Zhu G.H., Gray A.B., Patra H.K. (2022). Nanomedicine: Controlling nanoparticle clearance for translational success. Trends Pharmacol. Sci..

[105] Jacobson K.H., Gunsolus I.L., Kuech T.R., Troiano J.M., Melby E.S., Lohse S.E., Hu D., Chrisler W.B., Murphy C.J., Orr G. (2015). Lipopolysaccharide density and structure govern the extent and distance of nanoparticle interaction with actual and model bacterial outer membranes. Environ. Sci. Technol..

[106] Häffner S.M., Malmsten M. (2017). Membrane interactions and antimicrobial effects of inorganic nanoparticles. Adv. Colloid Interface Sci..

[107] Behera N., Arakha M., Priyadarshinee M., Pattanayak B.S., Soren S., Jha S., Mallick B.C. (2019). Oxidative stress generated at nickel oxide nanoparticle interface results in bacterial membrane damage leading to cell death. RSC Adv..

[108] Das B., Dash S.K., Mandal D., Ghosh T., Chattopadhyay S., Tripathy S., Das S., Dey S.K., Das D., Roy S. (2017). Green synthesized silver nanoparticles destroy multidrug resistant bacteria via reactive oxygen species mediated membrane damage. Arab. J. Chem..

[109] Yang Y., Fan S., Chen Q., Lu Y., Zhu Y., Chen X., Xia L., Huang Q., Zheng J., Liu X. (2022). Acute exposure to gold nanoparticles aggravates lipopolysaccharide-induced liver injury by amplifying apoptosis via ROS-mediated macrophage-hepatocyte crosstalk. J. Nanobiotechnol..

[110] Malik S., Muhammad K., Waheed Y. (2023). Emerging applications of nanotechnology in healthcare and medicine. Molecules.

[111] Tripathi D., Pandey P., Sharma S., Rai A.K., Manjunatha Prabhu B.H. (2024). Advances in nanomaterials for precision drug delivery: Insights into pharmacokinetics and toxicity. BioImpacts.

[112] John J. (2025). Advancements in nano-based drug delivery systems for therapeutics: A comprehensive review. RSC Pharm..

[113] Desai N., Rana D., Patel M., Bajwa N., Prasad R., Vora L.K. (2025). Nanoparticle therapeutics in clinical perspective: Classification, marketed products, and regulatory landscape. Small.

[114] Ewii U.E., Attama A.A., Olorunsola E.O., Onugwu A.L., Nwakpa F.U., Anyiam C., Chijioke C., Ogbulie T. (2025). Nanoparticles for drug delivery: Insight into in vitro and in vivo drug release from nanomedicines. Nano TransMed.

[115] Rosenblum D., Joshi N., Tao W., Karp J.M., Peer D. (2018). Progress and challenges towards targeted delivery of cancer therapeutics. Nat. Commun..

[116] Zhu L. (2021). Stimuli-Responsive Nanomedicine.

[117] Zelepukin I.V., Shevchenko K.G., Deyev S.M. (2024). Rediscovery of mononuclear phagocyte system blockade for nanoparticle drug delivery. Nat. Commun..

[118] Lu J., Gao X., Wang S., He Y., Ma X., Zhang T., Liu X. (2023). Advanced strategies to evade the mononuclear phagocyte system clearance of nanomaterials. Exploration.

[119] Song G., Petschauer J.S., Madden A.J., Zamboni W.C. (2014). Nanoparticles and the mononuclear phagocyte system: Pharmacokinetics and applications for inflammatory diseases. Curr. Rheumatol. Rev..

[120] Qie Y., Yuan H., Von Roemeling C.A., Chen Y., Liu X., Shih K.D., Knight J.A., Tun H.W., Wharen R.E., Jiang W. (2016). Surface modification of nanoparticles enables selective evasion of phagocytic clearance by distinct macrophage phenotypes. Sci. Rep..

[121] Salvati A., Pitek A.S., Monopoli M.P., Prapainop K., Bombelli F.B., Hristov D.R., Kelly P.M., Åberg C., Mahon E., Dawson K.A. (2013). Transferrin-functionalized nanoparticles lose their targeting capabilities when a biomolecule corona adsorbs on the surface. Nat. Nanotechnol..

[122] Danhier F., Ansorena E., Silva J.M., Coco R., Le Breton A., Préat V. (2012). PLGA-based nanoparticles: An overview of biomedical applications. J. Control. Release.

[123] Makadia H.K., Siegel S.J. (2011). Poly lactic-co-glycolic acid (PLGA) as biodegradable controlled drug delivery carrier. Polymers.

[124] Wang Y., Wang K., Zhao J., Liu X., Bu J., Yan X., Huang R. (2013). Multifunctional mesoporous silica-coated graphene nanosheet used for chemo-photothermal synergistic targeted therapy of glioma. J. Am. Chem. Soc..

[125] Ayobami A.O., Abimbola A.A. (2025). Advances in Nanoparticles as Drug Delivery Systems: A Review. Sci. Afr..

[126] Anselmo A.C., Mitragotri S. (2019). Nanoparticles in the clinic: An update. Bioeng. Transl. Med..

[127] Ventola C.L. (2017). Progress in nanomedicine: Approved and investigational nanodrugs. Pharm. Ther..

[128] Farah H., Kadhim-Abosaoda M., Mohaisen-Mousa H., Renuka Jyothi S., Priyadarshini-Nayak P., Bethanney Janney J., Singh G., Singh-Chauhan A., Kumar-Mishra M. (2026). Nanomedicine Strategies Against Biofilm-Associated Infections: Advances, Challenges, and Translational Barriers. MicrobiologyOpen.

[129] Xie Y., Liu H., Teng Z., Ma J., Liu G. (2025). Nanomaterial-enabled anti-biofilm strategies: New opportunities for treatment of bacterial infections. Nanoscale.

[130] Gao Y., Wang W., Yue X., Wang G., Zhang K., Wu C., Zhao Z., Huang Z., Zhang X. (2025). Inhalable nanoparticle-based delivery systems for the treatment of pulmonary infections: Status quo and barrier-overcoming strategies. Drug Deliv..

[131] Li S.-D., Huang L. (2008). Pharmacokinetics and biodistribution of nanoparticles. Mol. Pharm..

[132] Halwani A.A. (2022). Development of pharmaceutical nanomedicines: From the bench to the market. Pharmaceutics.

[133] Mülhopt S., Diabaté S., Dilger M., Adelhelm C., Anderlohr C., Bergfeldt T., Gómez de la Torre J., Jiang Y., Valsami-Jones E., Langevin D. (2018). Characterization of nanoparticle batch-to-batch variability. Nanomaterials.

[134] Abdullah M., Obayedullah M., Shuvo M.S.I., Khair M.A., Hossain D., Islam M.N. (2025). A review on multifunctional applications of nanoparticles: Analyzing their multi-physical properties. Results Surf. Interfaces.

[135] Sharmile N., Chowdhury R.R., Desai S. (2025). A comprehensive review of quality control and reliability research in micro–nano technology. Technologies.

[136] Agrahari V., Hiremath P. (2017). Challenges associated and approaches for successful translation of nanomedicines into commercial products. Nanomedicine.

[137] Kelly O.M., Hanna A.R., Byrne A.K., Green J.A., Perisse I.V., Wells K.D., Murray S.A., Xu J., Chen Y.E., Polejaeva I.A. (2026). Towards clinical translation of nanomedicines: Formulation scale-up and large animal models. Adv. Drug Deliv. Rev..

[138] Brem J., Cain R., Cahill S., McDonough M.A., Clifton I.J., Jiménez-Castellanos J.-C., Avison M.B., Spencer J., Fishwick C.W., Schofield C.J. (2016). Structural basis of metallo-β-lactamase, serine-β-lactamase and penicillin-binding protein inhibition by cyclic boronates. Nat. Commun..

